# Quantitative imaging mass spectroscopy reveals roles of heme oxygenase-2 for protecting against transhemispheric diaschisis in the brain ischemia

**DOI:** 10.3164/jcbn.17-136

**Published:** 2018-04-11

**Authors:** Shinichi Goto, Takayuki Morikawa, Akiko Kubo, Keiyo Takubo, Keiichi Fukuda, Mayumi Kajimura, Makoto Suematsu

**Affiliations:** 1 Department of Biochemistry, Keio University School of Medicine, 35 Shinano-machi, Shinjuku-ku, Tokyo 160-8582, Japan; 2 Department of Cardiology, Keio University School of Medicine, 35 Shinano-machi, Shinjuku-ku, Tokyo 160-8582, Japan

**Keywords:** HO-2, IMS, microcirculation, metabolome analysis, stroke

## Abstract

Carbon monoxide-generating heme oxygenase-2 is expressed in neurons and plays a crucial role for regulating hypoxic vasodilation through mechanisms unlocking carbon monoxide-dependent inhibition of H_2_S-generating cystathionine β-synthase expressed in astrocytes. This study aims to examine whether heme oxygenase-2 plays a protective role in mice against stroke. Focal ischemia was induced by middle cerebral artery occlusion. Regional differences in metabolites among ipsilateral and contralateral hemispheres were analysed by quantitative imaging mass spectrometry equipped with an image-processing platform to optimize comparison of local metabolite contents among different animals. Under normoxia, blood flow velocity in precapillary arterioles were significantly elevated in heme oxygenase-2-null mice vs controls, while metabolic intermediates of central carbon metabolism and glutamate synthesis were elevated in the brain of heme oxygenase-2-null mice, suggesting greater metabolic demands to induce hyperemia in these mice. In response to focal ischemia, heme oxygenase-2-null mice exhibited greater regions of ischemic core that coincide with notable decreases in energy metabolism in the contralateral hemisphere as well as in penumbra. In conclusion, these findings suggest that heme oxygenase-2 is involved in mechanisms by which not only protects against compromised energy metabolism of the ipsilateral hemisphere but also ameliorates transhemispheric diaschisis of the contralateral hemisphere in ischemic brain.

## Introduction

Heme oxygenase (HO) is a mono-oxygenase utilizing heme and molecular oxygen (O_2_) as substrates to generate biliverdin-IXα and carbon monoxide (CO). HO-1 is inducible under stress conditions, while HO-2 is constitutive. In liver, testes, and brain, HO-2 constitutes a major fraction of CO generation *in vivo*.^(1)^ In the liver, HO-2 expressed mainly in hepatocytes continuously generates CO to constitutively relax sinusoids through its relaxing action on hepatic stellate cells;^(2)^ such events guarantee ample blood flow for this organ to detoxify heme. In testes, CO generated by HO-2 and stress-inducible HO-1 causes apoptosis of spermatocytes to eliminate abnormally proliferated cells in the organ.^(3)^ But roles of CO generated from HO-2 in brain remained unknown.

We have revealed that CO generated from neurons expressing HO-2 serves as a tonic regulator of neurovascular units of the brain through its inhibitory action on cystathionine β-synthase (CBS); upon global hypoxia induced by 10% O_2_ inhalation, CO released from neural HO-2 in the presence of O_2_ as a substrate is decreased and unlock CBS in astrocytes to increase H_2_S, a vasorelaxing gas mediator. Thus, HO-2/CBS system serves as an important mechanism for hypoxia-induced vasorelaxation in neurovascular units;^(4)^ such a crucial role of neural HO-2 in hypoxic vasorelaxation of cortical microcirculation might benefit amelioration of damages in focal ischemia or stroke, but remained to be examined. This study aimed to examine whether expression of HO-2 in brain plays a protective role against focal ischemia in a middle cerebellar artery occlusion (MCAO) model.

The current results suggest that, HO-2 benefits for protection against compromised energy metabolism of the ipsilateral hemisphere. Furthermore, this enzyme plays a crucial role for ameliorating transhemispheric diaschisis of the contralateral hemisphere in the MCAO-induced stroke model of mice.

## Materials and Methods

### Animals

In this study, 2 strains of mice, HO-2-null on the C57BL/6J background and its littermate were utilized.^(5)^ Mice heterozygous for the disruption in the HO-2 gene were the generous gift from Eiichiro Nagata (Department of Neurology, Tokai University School of Medicine). They were bred at Keio University School of Medicine and fed *ad libitum* a standard CE-2 diet (Clea Japan, Tokyo, Japan). Genotypes were examined by polymerase chain reaction (PCR) of tail genomic DNA (see Supplemental Table 1* for primers used for genotyping). All experiments were approved by the Animal Care and Utilization Committee of Keio University School of Medicine (permission number 08040).

### Measurements of vessel diameters and blood flow velocities

Male mice (described in the animal section, 8–10 weeks-old, 22–26 g, Clea Japan) were anesthetized with an intraperitoneal injection of α-chloralose (60 mg/kg) and urethane (600 mg/kg). All mice were tracheotomised with a polyethylene catheter (PE-90, Intragenic, Clay Adams, NY) and allowed to breathe spontaneously under monitoring rectal temperature.

**Fig. 1:**
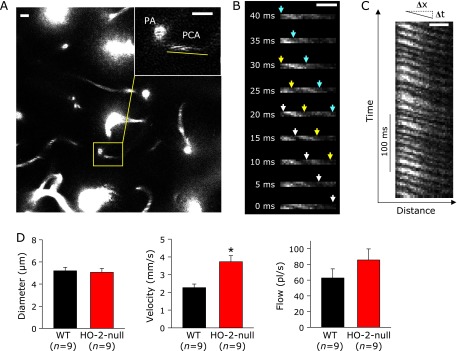
Differences of baseline velocity of red blood cell in precapillary arteriole in the brain between wild-type (WT) and HO-2-null mice. (A) A representative image of cortical microvessels visualized by 2-photon laser confocal microscopy. Inset in a yellow square indicates a precapillary arteriole (PCA) branching from the penetrating arteriole (PA). The right upper panel is the magnified picture of the inset. Bar = 10 µm. Time-dependent scanning on precapillary arteriole was carried out on the region indicated with the yellow line. (B) Representative sequential images obtained from the precapillary arteriole as a function of time for scanning (every 5 ms). Passage of red blood cells were detected as blank signals of Qdot fluorescence as indicated by white, yellow and blue arrows that indicate movement of 3 different blank densities resulting from circulating erythrocytes. Bar = 10 µm. (C) A series of PCA images compiled as a function of time (Y-axis) to calculate microvascular velocities. The velocities were measured by calculating the slope of the black line in which the ratio of Δx vs Δt gives regional velocities of erythrocytes. Based on these pictures, at least 5 PCAs were chosen to determine the velocity to calculate the average velocity. At least 2 different regions were chosen in a single animal experiment to determine one average value of the PCA velocity. Bar = 10 µm. (D) Comparison of diameters, red blood cell velocity and calculated flow volume in the precapillary arteriole at baseline. Although the diameters were not different between the two groups (*p* = 0.76), HO-2-null mice exhibited significantly higher velocity in precapillary arterioles. The calculated flow volume displayed 50% insignificant increase in HO-2-null mice (*p* = 0.13). *p<0.05 compared with the data of wild-type mice (WT). Values are mean ± SE of individual average PCA velocity collected from 9 separate experiments.

Blood vessels were visualized with an upright-type 2-photon laser microscope (BX61WI, Olympus, Tokyo, Japan) equipped with a mode-locked Titanium-Sapphire laser system (Chameleon Vision II, Coherent, Tokyo, Japan) that could achieve 140-fsec pulse width and 80-MHz repetition rate with 17-W pump power at 920 nm for laser excitation. To avoid brain herniation and to keep gas-tight conditions, thinned skull preparation was used to achieve microscopic transparency of the skull.^(6)^ A small bolus of 15 µl of Qdot^®^ 655 nanocrystals and 85 µl of saline were injected slowly to visualize the cerebral microvasculature.^(7)^ We identified penetrating arterioles from the arteries on the cerebral surface and traced along a penetrating arteriole to locate branched precapillary arterioles at a depth of ≈100 µm (Fig. 1A). Line-scanning method assisted with Fluoview^TM^ software was applied to measure the velocity of red blood cells running through a target vessel of interest where the cell was visualised as a blank signal between adjacent Q-dot-positive plasma compartments.^(8,9)^ Flow volume was calculated for each precapillary arteriole using the following formula:

*Flow volume = πr^2^ × V_r_*

where *r* is the radius and *V_r_* is the red blood flow velocity measured in the precapillary arteriole.

Laser-confocal images were acquired with a scanning unit (FV1000, Olympus, Tokyo, Japan) using Fluoview^TM^ software (FV10-ASW, ver. 2.0, Olympus) and a 25 × objective (XLPLN25 × WMP, NA 1.05). Fluorescence emission was detected by an external photomultiplier tube (R3896, Hamamatsu Photonics, Hamamatsu, Japan) after passing through an infrared blocking filter (685-nm cut off) and an emission filter (641–675 nm for Qdot^®^655).

### Middle cerebral artery occlusion (MCAO) model

 Male mice (described in the animal section, 8–10 weeks-old, 22–26 g, Clea Japan) were anesthetized with isoflurane (4% for induction, 1.5–2% for maintenance) with the rectal temperature monitored. A focal brain ischemia was induced for 60 min by advancing a 6-0 nylon suture with its tip rounded to occlude the origin of a middle cerebral artery (MCA) as described previously.^(10,11)^

Occlusion was confirmed by the laser Doppler (ALF21, Advance, Tokyo, Japan).

### *In-situ* freezing procedures for collecting brain tissues

To protect from enzymatic degradation of metabolites such as phosphorylated adenylates, we employed the *in-situ* freezing method^(12)^ which enables suspension of metabolic processes by rapidly lowering the tissue temperature while maintaining blood flow and oxygenation during the freezing process. To do so, mice were anesthetized with isoflurane according to our previous method.^(4,10)^ Tip of the head was dipped into liquid nitrogen with great care not to immerse the nose. Frozen brains were dissected with a surgical knife in a refrigerated box at −30°C.

### Metabolome analysis of brain tissue

A 2-mm thick frozen coronal section adjacent to the section used for the imaging mass spectrometry (IMS) analysis was used for metabolome analyses as described previously.^(4,10,13–15)^ Experiments were performed using an Agilent CE Capillary Electrophoresis System (CE/ESI/MS) equipped with an air pressure pump, an Agilent 1200 series MSD^®^ mass spectrometer and an Agilent 1200 series isocratic high performance liquid chromatography pump, a G1603A Agilent CE/MS adapter kit, and a G1607A Agilent CE/MS sprayer kit (Agilent Technologies, CA).

### Imaging mass spectrometry

The visualization of distributions of metabolites were done utilizing the matrix assisted laser desorption/ionization (MALDI) IMS method.^(4,10,13–16)^ The details of the method for performing the MALDI-IMS in brain tissues were described previously.^(10)^ In brief, coronal sections with a 10-µm thickness were cut using a cryo-microtome (CM3050s; Leica Microsystems, Wetzlar, Germany). The sections were thaw-mounted on indium tin oxide (ITO)-coated glass slide (No. 8237001, Bruker Daltonics, Billerica, MA). Freshly prepared 9-aminoacridine (25 mmol/L solution in 80% (*v/v*) ethanol) was sprayed over the samples using an airbrush (PS-270, Mr. Hobby, Tokyo, Japan). The samples were then subjected to raster scanning for MALDI-IMS. Mass spectrometric imaging experiments were performed using the negative ionization mode. The data were acquired using MALDI mass spectrometer (AXIMA Performance MALDI TOF/TOF, and AXIMA Resonance MALDI QIT TOF/TOF; Shimadzu Corporation, Kyoto, Japan) with 337-nm N2 laser, at 10 Hz, operated in the linear negative mode with a scan pitch of 200 µm according to our previous experiment.^(10)^ Mass spectra were obtained with the following conditions: accelerating voltage of 20 kV, scanning mass range of 1–2,000 Da, and “pulsed extraction mass” was set to 1,000 Da, respectively. All imaging data were acquired in a vacuum condition (8.0 10 5 Pa). The number of laser shots per spot was set to 200 with a roaming function that enables to obtain averaged data from each spot, avoiding the effects of spotty matrix.

### Determination of apparent contents of metabolites by quantitative imaging mass spectrometry

To construct apparent content maps for a target metabolite of interests, quantitative imaging mass spectrometry (Q-IMS) was used according to our previous studies.^(4,10,13–15)^ Briefly, Q-IMS is a method to estimate apparent contents of metabolite within a region of interest by combining spatial distribution determined by MALDI-IMS with absolute quantity determined by CE/ESI/MS using adjacent tissue sections. To do so, mass signal intensities of a metabolite acquired with MALDI-IMS were converted to apparent contents of a metabolite expressed in an absolute term (tissue content in nmol/g tissue). Apparent content of a metabolite at the i^th^ spot of tissue (*C_i_*) was estimated as follow:

*C_i_ = C’ × I_i_/Ī*

where *C’* denotes the metabolite content of tissue from a contralateral hemisphere determined by the CE/MS, *I_i_* is the maximum intensity among mass spectra in a specified range at the i^th^ spot, and *Ī* is the median of maximum intensities of a metabolite from all the spots in a contralateral hemisphere. To perform unbiased analysis, we newly developed a platform named Rapid Image Contrast Enhancement (RICE), which enables region specific analysis and automatic colored image production from Q-IMS data.

### Analysis on patency of microvessels in cortex of ischemic brain

Male mice (described in the “Animals” section, 8–10 weeks-old, 22–26 g, Clea Japan) were anesthetized with isoflurane (4% for induction, 1.5–2% for maintenance). Rectal temperature was monitored throughout the experiment. A focal brain ischemia was induced for 60 min as described in the “MCAO model” section by advancing a 6-0 nylon suture into the MCA. To visualize the hypoxic area, hypoxyprobe-1 kit (Cosmo Bio Co., Ltd, Tokyo, Japan) using pimonidazole as a hypoxic probe was used. Intraperitoneal injection of 60 mg/g pimonidazole was performed at 30 min after the induction of focal ischemia. To visualize perfused microvessels, mice were further intravenously injected 4 mg/g FITC labelled lectin.

Mice were decapitated after completion of 60-min ischemia. Coronal sections of the brain with a 60-µm thickness were cut using a cryo-microtome (CM3050s; Leica Microsystems, Wetzlar, Germany). The sections were stained with CD31. Images were acquired with a scanning unit (FV1000, Olympus) using Fluoview^TM^ software (FV10-ASW, ver. 2.0, Olympus) and a 25 × objective (XLPLN25 × WMP, NA 1.05).

In our images, a single pixel represented 2.5 µm. The majority of the diameters of microvessels in the cortex were within 4 µm, and covered a 1-pixel width. Thus, the total lengths of microvessels of this width were measured by counting up the numbers of pixels that displayed positive CD31 fluorescence. Microvessels with greater diameters than 5 µm (>2 pixels), were determined manually by ImageJ software. The cross-sectional areas of hypoxic regions were measured by summing up all the pixels indicating positive pimonidazole staining within the cortex. Since the border of positive- and negative-pimonidazole staining could be easily determined in the cortex, such area of hypoxia was manually selected by ImageJ software. Patency of microvessels were measured by calculating the total lengths of lectin-positive microvessels. In these measurements, lengths of the microvessels were calculated by the method identical to that used for lengths of CD31-positive microvessels as described above. Such values of microvascular patency were standardized by dividing these values by the lengths of CD31-positive microvessels.

## Results

### Blood flow velocities of precapillary arterioles were elevated at baseline in HO-2-null mice

Vessel diameters and velocities of red blood cells were compared between wild-type (WT) and HO-2-null mice. Representative images illustrating precapillary arterioles branching from penetrating arterioles were captured in and around 100-µm depth from the pia through the thinned skull preparation window of mice^(6)^ (Fig. 1A); 2-dimensional laser scanning at this depth enabled us to visualize neurovascular units of the cortex.^(4)^ Diameters of precapillary arterioles were measured from these images. Velocity of red blood cells in precapillary arterioles were measured using the line-scan method (Fig. 1B and C). While the diameters of precapillary arterioles were not different between the two groups, HO-2-null mice exhibited higher velocity of red blood cells in precapillary arterioles (Fig. 1D). These results suggest increased blood flow in cortical neurovascular units at baseline levels in HO-2-null mice.

### Enhancement of glycolysis, pentose phosphate pathway and glutamate synthesis were observed in HO-2-null mice at baseline

**Fig. 2:**
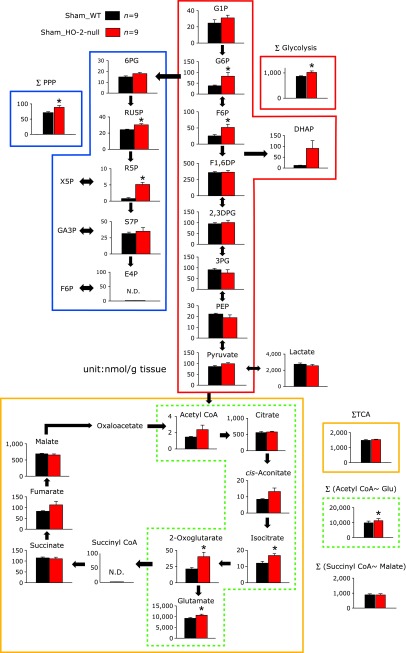
Differences in baseline levels of metabolites in brain between sham-operated wild-type (WT) and HO-2-null mice under normoxia. Comparisons of metabolites in the sham-operated contralateral hemisphere of the brain between wild-type (WT) and HO-2-null mice. Total summation of metabolic intermediates belonging to glycolysis (solid red lines, ∑ glycolysis) and pentose phosphate pathway (solid blue lines, ∑ PPP) were significantly elevated in HO-2-null mice, while the sum of metabolites of TCA cycle (solid yellow lines, ∑ TCA) was unchanged. To be noted, the sum of metabolites leading to glutamate synthesis (dotted green lines, ∑ acetyl CoA~Glu) was significantly elevated in HO-2-null mice. On the other hand, the rest of the TCA intermediates (∑ succinyl CoA~malate) was not different between HO-2-null mice and wild type mice. G1P, glucose-1-phosphate; G6P, glucose-6-phosphate; F6P, fructose-6-phosphate; F1,6DP, fructose-1,6-biphosphate; 2,3DPG, 2,3-diphophoglycerate; 3PG, 3-phosphoglycerate; PEP, phosphoenolpyruvate; DHAP, dihydroxyacetone phosphate; 6PG, 6-phosphogluconate; RU5P, ribulose-5-phosphate; R5P, ribose-5-phosphate; S7P, sedoheptulose-7-phosphate; E4P, erythrose-4-phosphate; X5P, xylulose-5-phosphate, GA3P, glyceraldehyde-3-phosphate; TCA, tricarboxylic acid. *p<0.05 compared with WT using unpaired Student’s *t* test. Values are mean ± SE of 9 separate experiments, and the unit of all data is nmol/g tissue.

Since the blood flow in cortical neurovascular units are controlled by metabolic demands of neurons,^(17)^ the observation that baseline blood flow was higher in the HO-2-null mice lead us to hypothesis that nutritional demands of neurons might be elevated in HO-2-null mice. To test the hypothesis, we attempted to compare differences of the baseline metabolism under normoxia between wild-type and HO-2-null mice, and performed metabolome analyses using the contralateral hemisphere of sham-operated mice (Fig. 2). The comparison revealed significant elevation of the sum of metabolic intermediates of glycolysis (highlighted in red lines) and pentose phosphate pathway (highlighted in blue lines) in HO-2-null mice. The total amounts of TCA cycle metabolites were apparently comparable between the two groups. However, the sum of TCA cycle intermediates for synthesising glutamate (highlighted in dotted green lines) was significantly elevated in HO-2-null mice. These results suggested increased flux to the glycolysis, pentose phosphate pathway and glutamate synthesis at baseline.

### Impaired energy metabolism was observed in the contralateral of HO-2-null mice after focal ischemia

**Fig. 3:**
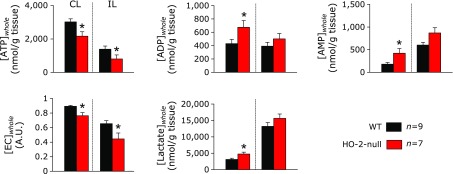
Comparisons of phosphonucleotide contents in ipsilateral and contralateral hemispheres of MCA-occluded brain between wild-type (WT) and HO-2-null mice. CL, contralateral hemisphere; IL, ipsilateral hemisphere. ATP contents and EC of HO-2-null mice were significantly lower than those of the wild-type mice in both CL and IL regions. To note is that contents of ADP, AMP and lactate were greater in the contralateral hemisphere of HO-2-null mice than in that of the wild-type mice, while those in in the ipsilateral hemisphere were not different between the two groups. ATP, adenosine-triphosphate; ADP, adenosine-diphosphate; AMP, adenosine monophosphate; EC, energy charge; MCAO, middle cerebral artery occlusion. *p<0.05 compared with WT using unpaired Student’s *t* test. Values are mean ± SE of given numbers of separate experiments.

HO-2 has been reported to play protective roles in brain during global hypoxia.^(4)^ However, it is unknown weather HO-2 could play similar protective roles in pathological conditions such as stroke. To test this hypothesis, energy metabolites and lactate were compared between the wild-type and HO-2-null mice after 60-min focal ischemia induced by MCAO (Fig. 3). In the ipsilateral hemisphere, adenosine triphosphate (ATP) was significantly decreased in HO-2-null mice as compared with the wild-type mice. Under these circumstances, adenosine diphoshate (ADP) and adenosine monophosphate (AMP) were unchanged. As a result, energy charge (EC) became significantly low in the HO-2-null mice. In the contralateral hemisphere, however, ATP and EC were significantly decreased in HO-2-null mice as compared with the wild-type mice. Surprisingly, while lactate in the ipsilateral hemisphere was elevated comparably in the wild-type and HO-2-null mice, that in the contralateral hemisphere was significantly higher in HO-2-null mice than in the wild-type mice. These results indicate that focal ischemia leads to the metabolic alterations in remote regions, when HO-2 is genetically disrupted.

**Fig. 4:**
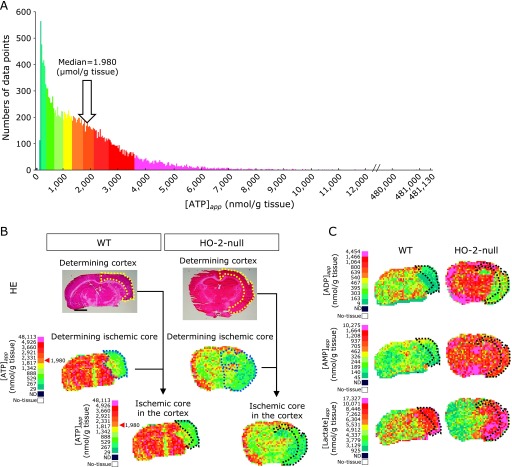
Extraction of ischemic core boundaries by mapping apparent ATP contents ([ATP]_app_) and comparison with spatial distribution of [ADP]_app_, [AMP]_app_ and [Lactate]_app_. (A) Histogram of apparent ATP contents ([ATP]_app_) at individual pixels in quantitative imaging mass spectrometry. Data of histogram were obtained by utilizing RICE on MCAO models of wild-type (*n* = 9) and HO-2-null mice (*n* = 7). Data of content values at the individual pixels are collected from both wild-type (*n* = 9) and HO-2-null mice (*n* = 7), and their frequency distribution was plotted as a function of apparent [ATP]_app_. Since the histogram did not follow Gaussian distribution, the median value of the histogram was defined as the threshold to determine the ischemic core regions. Namely, the pixels showing less than 1.98 µmol/g tissue of [ATP]_app_ were defined as ischemic core regions. (B) The process of determining ischemic core region in the cortex from HE stained tissue, and IMS data of the wild-type and HO-2-null mice undergoing MCAO. The cortex was determined using the Haematoxylin-Eosin (HE)-stained tissues (indicated with yellow dotted lines). Raw data of ATP in IMS were converted to an apparent content map of ATP ([ATP]_app_) through being processed by color coding by RICE. Ischemic core (indicated with blue dotted lines) were defined as pixels indicating the signals less than median of all data points (1.98 µmol/g tissue). Ischemic core region in the cortex (indicated with black dotted lines) was determined by taking the intersection of the ischemic core and the cortex. (C) Spatial distribution of ADP, AMP and lactate in ischemic core regions in the cortex of the wild-type and HO-2-null mice. Measurements of individual metabolites in the ischemic core of the cortex were carried out using the regions defined in Panel B. ATP, adenosine triphosphate (*m/z* 505.98); ADP, adenosine diphosphate (*m/z* 426.02); AMP, adenosine monophosphate (*m/z* 346.08). Lactate (*m/z* 89.03). Bar = 2.0 mm.

To examine regional differences in energy metabolism among the ischemic core, penumbra and non-ischemic regions of brains between the wild-type and HO-2-null mice, we attempted to analyse distribution of pixel-based apparent ATP contents ([ATP]_app_) in all brain samples containing 9 wild-type and 7 HO-2-null mice. The histogram of [ATP]_app_ obtained from these 16 samples showed a non-Gaussian distribution (Fig. 4A), in which the median value of all the data was 1.98 µmol/g tissue. This value was approximately 50% of [ATP]_app_ values in the intact brain treated with in-situ freezing.^(10)^ We thus used this value as the threshold of [ATP]_app_, and the pixels indicating the values less than the median were considered to belong to ischemic core regions (Fig. 4B). Since some variations of MCA perfuse the hippocampus in mouse brain, we focused only on the cortex. As seen in Fig. 4C, we analysed differences in alterations in other metabolites (e.g., ADP, AMP, and lactate) in the ischemic core regions of the cortex (the area surrounded by dotted black lines), which were defined as the region displaying [ATP]_app_ values less than 1.98 µmol/g tissue.

### Impairment of energy metabolism in penumbra and the cortex of contralateral hemisphere upon focal ischemia in HO-2-null mice

**Fig. 5:**
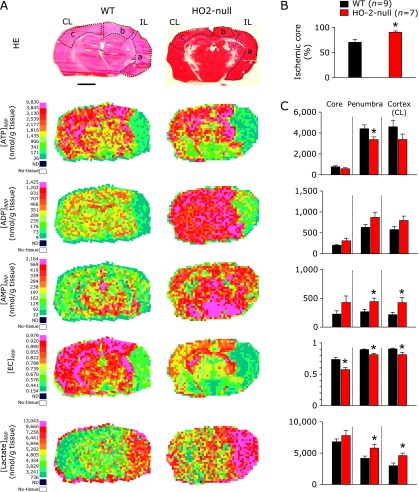
Comparisons of metabolites in the penumbra and in the cortex of contralateral hemisphere upon focal ischemia in HO-2-null mice revealed with region specific analysis. (A) Comparisons of metabolites in specific regions of MCAO-treated brain, such as ischemic core (a), penumbra (b) and contralateral cortex (c). All metabolites were imaged from a single brain slice per sample. The brain slice was stained with Haematoxylin-Eosin (HE) staining after imaging the metabolites. Mapping of energy charges [EC]_app_ per each pixel were calculated according to the definition of the formula and the apparent contents of ATP, ADP ([ADP]_app_) and AMP ([AMP]_app_) in individual pixels. (B) Comparison of the size of ischemic core in the cortex between the wild-type and HO-2-null mice. Ischemic core was defined as the pixel where [ATP]_app_ values was lower than the median value (1.98 µmol/g tissue) obtained from 9 wild-type and 7 HO-2-null mice according to the data shown in Fig. 4. HO-2-null mice exhibited significantly greater areas of ischemic core as compared with wild-type mice. (C) Region-specific comparison of apparent contents of ATP, ADP, AMP, and lactate and energy charge and (EC) values between MCAO model of wild-type and HO-2-null mice. In the ischemic core, HO-2-null mice showed lower EC, while no other significant differences were observed. In the penumbra, HO-2-null mice had lower EC and [ATP]_app_, and higher contents of AMP and lactate. In the cortex of the contralateral hemisphere, HO-2-null mice exhibited lower EC and higher contents of AMP and lactate. The region outlined with the dotted line indicates ischemic core (a), penumbra (b), and the cortex of contralateral hemisphere (c). IL, ipsilateral; CL, contralateral. Bar = 2.0 mm. ATP, adenosine-triphosphate (*m/z* 505.98); ADP, adenosine-diphosphate (*m/z* 426.02); AMP, adenosine-monophosphate (*m/z* 346.08); EC, energy charge. Lactate (*m/z* 89.03). *p<0.05 as compared with WT using unpaired Student’s *t* test. Values are mean ± SE of given numbers of separate experiments.

Figure 5 illustrates content maps of ATP, ADP, AMP and lactate that displays spatial distribution of their apparent contents in focal ischemic brain of wild-type and HO-2-null mice. As seen in Fig. 5A, we examined regional contents of these metabolites in 3 different cortical regions of brain; ischemic core, penumbra in the ipsilateral, and apparently intact cortical region in the contralateral hemisphere. In this model, we compared differences in ATP-decreasing regions between the wild-type and HO-2-null mice. As seen in Fig. 5B, disruption of HO-2 gene caused a significant elevation of the cross-sectional areas of ischemic core in the cortex, which was calculated using the following formula to compensate for the swelling effect.^(18)^

*Ischemic core ratio (%) = (C_i_ *–* N_i_)/C_i_*

where *N*_i_ denotes the non-ischemic-core region defined as the area with ATP higher than the threshold in the cortex of ipsilateral hemisphere determined by the Q-IMS and C*_i_* is area of cortex in contralateral hemisphere.

To further analyse the regional changes in metabolites, we compared the data collected from HO-2-null mice with wild-type mice (Fig. 5C). In the ischemic core, no significant differences were seen between the 2 groups, while [EC]_app_ values became significantly lower in the HO-2-null mice. In the penumbra region, HO-2-null mice displayed lower [ATP]_app_ and higher [AMP]_app_ that resulted in lower [EC]_app_. To be noted was [lactate]_app_ values were significantly increased in HO-2-null mice as compared with those in the wild-type mice. These results suggest that HO-2 gene is necessary to improve the quality of penumbra regions in this stroke model of mice.

Serendipitous observation revealed by imaging mass spectrometry was the fact that, in the HO-2-null mice, the contralateral hemisphere exhibited higher [AMP]_app_ with lower [EC]_app_, and significant elevation of lactate. Functional alterations in the remote regions of the ischemic brain is known as diaschisis.^(19–23)^ Remote effects in the contralateral hemisphere distant from the site of original focal cerebral ischemia in the ipsilateral hemisphere are referred to as “transhemispheric diaschisis” in our study. Our current method using Q-IMS revealed that multiple metabolites in the contralateral hemisphere were changed in HO-2-null mice when they were exposed to focal ischemia. Collectively, these results suggest that HO-2 plays a crucial role for ameliorating transhemispheric diaschisis in the MCAO-induced stroke model.

### Impaired microvascular patency in HO-2-null mice undergoing focal ischemia

**Fig. 6:**
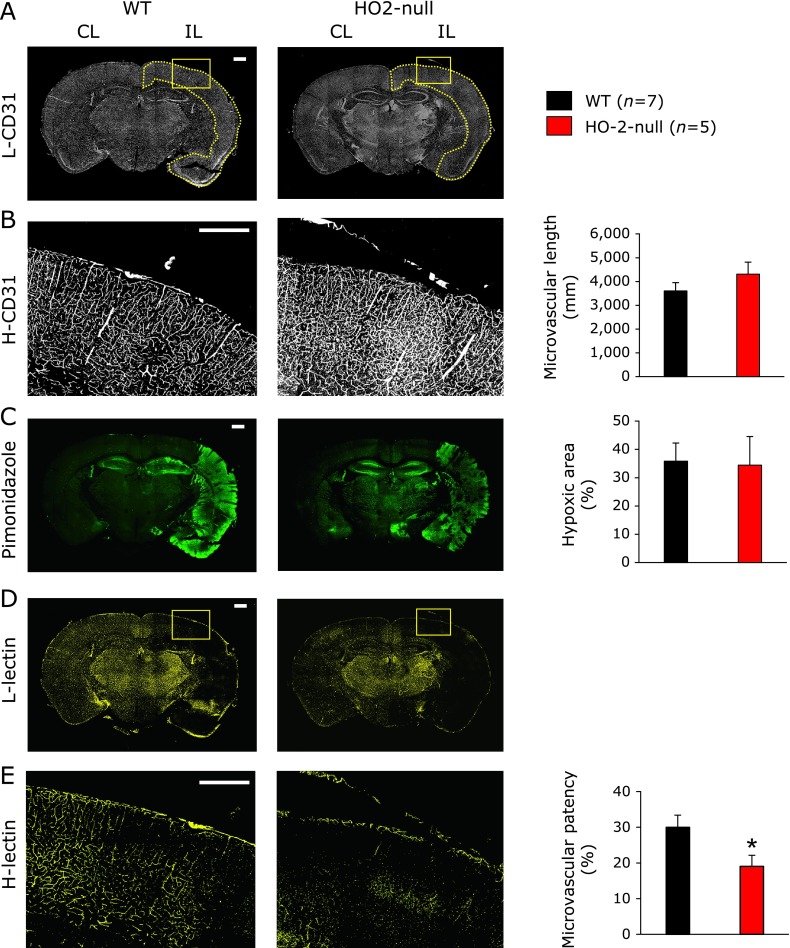
Differences in microvascular patency during MCAO-induced focal ischemia between wild-type and HO-2-null mice. (A) Representative micro-fluorographic images of CD31 as a marker of microvascular endothelium visualized at low-power (L-CD31) magnification. The dotted yellow line indicates the cortex of ipsilateral hemisphere. (B) Images of CD31 staining at high-power (H-CD31) magnification in the same slice. H-CD31 images were used for quantifying microvascular length. Mean ± SE of given numbers of separate experiments. Bars = 500 µm. (C) Representative images showing hypoxic regions judged by pimonidazole staining. Boundaries showing strong fluorescent signals were manually extracted in the ipsilateral hemisphere of the brain slices to determine hypoxic areas. Mean ± SE of given numbers of separate experiments. (D, E) Representative pictures collected by low- and high-power representative images of lectin staining, respectively. Since lectin was injected 30-min after focal ischemic procedure (60-min MCAO), the lectin labelling indicates the visualization of perfused microvessels. Mean ± SE of given numbers of separate experiments. *p<0.05 as compared with the values in the wild-type group using unpaired Student’s *t* test. Note that the baseline microvascular density (CD31) and the area of hypoxic regions were comparable between the wild-type and HO-2-null mice, while the microvascular patency was significantly lower in the HO-2-null mice than in the wild-type mice.

In our current experiments, the energy metabolisms were not only impaired in the penumbra but was also jeopardized in the contralateral hemisphere in HO-2-null mice. These findings, combined with the previous report that HO-2-null mice display impaired vasodilation in response to hypoxia,^(4)^ lead us to hypothesize that HO-2 plays important roles in vasodilation also during focal ischemia. To test this hypothesis, we measured and compared the total microvascular lengths, hypoxic region and perfused microvascular density in the cortex of ipsilateral hemisphere (indicated with the dotted yellow line) after 60-min focal ischemia (Fig. 6). The analysis on total vascular lengths in the cortex of ipsilateral hemisphere is shown in Fig. 6A. There were no differences between the HO-2-null and the wild-type mice. The total size of hypoxic regions measured by summing up the numbers of pixels showing pimonidazole signals were not different (Fig. 6B) between the 2 groups. However, the microvascular patency, were lower in HO-2-null as compared to wild-type mice (Fig. 6C). These results suggest that HO-2-null mice display impaired microvascular patency in the cortex in response to hypoxia caused by focal ischemia in our model.

## Discussion

In the present study, we have demonstrated protective roles played by HO-2 against stroke-induced metabolic impairment of mouse brain. The gene disruption of HO-2 does not only result in compromised energy metabolism in the ipsilateral hemisphere, but also cause transhemispheric diaschisis in the contralateral hemisphere in the MCAO-induced stroke model in mice.

While being a marginally significant elevation, the baseline flow volume of pre-capillary arterioles displayed approximately 50% increase in HO-2-null mice (*p* = 0.13, Fig. 1). HO is known to catalyse oxidative heme degradation that resultantly generates biliverdin-XIα and carbon monoxide (CO).^(1,2,24)^ Our previous study provided evidence that, under normoxia, endogenous CO derived from HO-2 expressed in neurons, blocks CBS, an H_2_S-generating enzyme expressed in astrocytes, to modulate neurovascular units.^(4)^ On global hypoxia, in which mice are exposed to 10% O_2_, the HO-2-involved mechanism is unlocked through a fall in O_2_-derived CO that resultantly triggers microvascular dilation. Lock of such an adaptive vascular response in HO-2-null mice compromises the brain’s ability to maintain ATP levels and the energy charge.^(4)^ Considering that HO-2 gene disruption causes approximately 50% drop of the baseline CO contents in the mouse brain, endogenous CO accounts for the baseline modulator of microvascular blood flow in the brain cortex. Since microvascular blood flow is regulated by regional metabolic demands for maintaining energy metabolism in brain,^(25,26)^ we examined differences in metabolomics of the baseline conditions under normoxia between wild-type and HO-2-null mice. Surprisingly, HO-2-null mice exhibited significant increases in total amounts of glycolytic intermediates and those of TCA intermediates responsible for the delivery of glutamate, a major neurotransmitter that is mainly synthesized through glucose oxidation.^(27)^

A rise in the blood flow velocity in HO-2-null mice (Fig. 1D) led us to hypothesize that HO-2 gene disruption might cause activation of O_2_ consumption and thereby up-regulate glucose oxidation through glycolysis and TCA cycle in brain, suggesting that constitutive HO-2 expression in brain limits the basal glucose oxidation in physiologically oxygenated brain. While the whole mechanisms by which HO-2 modulates glucose oxidation in brain remain unknown, several possibilities should be considered: First, the increased blood flow in HO-2-null mice may provide excessive glucose and O_2_ to the tissue, resulting in enhanced glycolysis driven by increased substrates. Secondly, roles of H_2_S and CO-sensitive CBS might play a role. Recent studies suggest that sulfhydration of glyceraldehyde-3-phosphate dehydrogenase (GAPDH) by H_2_S enhances its catalytic function.^(28)^ Our previous studies provided evidence that physiologic levels of CO inhibit CBS and thus reduces the production of H_2_S.^(4,29)^ Collectively with these reports, it is not unreasonable to speculate that HO-2 gene disruption might cause endogenous CO suppression to activate H_2_S-generating CBS and thereby increase the catalytic activity of GAPDH. Finally, inhibitory effects of CO on cytochrome *c* oxidase in mitochondria should be taken into accounts.^(4,30)^ Indeed, our results showed a significant elevation of metabolic intermediates in TCA cycle for synthesizing glutamate, being in good agreement with a hypothesis that HO-2-derived CO directly binds to cytochrome *c* oxidase to partially suppress its catalytic activities of electron transport system and TCA cycle. These lines of contexts led us to suggest that HO-2-derived CO may play protective roles for limiting unnecessary acceleration of glucose oxidation to control metabolic tolerance against local ischemia and transhemispheric diaschisis.

Although mechanisms of diaschisis remains unknown, to our knowledge, this is the first report showing that transhemispheric diaschisis occurs in 60-min MCAO in HO-2-null mice. In our previous studies, we observed no metabolic derangement in contralateral hemisphere of wild-type mice treated with identical procedures of 60-min MCAO-induced focal ischemia.^(10)^ The current results suggest that HO-2 is necessary to prevent transhemispheric diaschisis in the contralateral hemisphere of the brain exposed to 60-min MCAO. In summary, HO-2 protects against focal ischemic changes in stroke. Gene disruption of HO-2 does not only compromise cortical microvascular patency and energy metabolism in the ipsilateral hemisphere but also results in transhemispheric diaschisis in the contralateral hemisphere. Although mechanisms by which lack of HO-2 causes metabolic derangement in the contralateral hemisphere remain unknown, these results shed light on a potential paradigm shift of treatment of stroke from conventional management of local vascular re-canalization towards global management including control of transhemispheric diaschisis as a whole brain.

## Appendices*

## Abbreviations

ADP: adenosine diphosphate
AMP: adenosine monophosphate
ATP: adenosine triphosphate
CBS: cystathionine β-synthase
CE/MS: capillary electrophoresis mass spectrometry
CE/ESI/MS: capillary electrophoresis–electrospray ionization mass spectrometry
CO: carbon monoxide
CSV: comma separated value
HO: heme oxygenase
IMS: imaging mass spectrometry
MALDI: matrix assisted laser desorption/ionization
MCA: middle cerebral artery
MCAO: middle cerebral artery occlusion
PCR: polymerase chain reaction
Q-IMS: quantitative imaging mass spectrometry
RICE: rapid image contrast enhancement
WT: wild-type

## Supplementary Material

Supplemental Table 1

Appendices
