# Supplementary material for: Quantitative imaging mass spectroscopy reveals roles of heme oxygenase-2 for protecting against transhemispheric diaschisis in the brain ischemia
Source: J Clin Biochem Nutr. 2018 Apr 11;63(1):70–9. doi: 10.3164/jcbn.17-136 (PMC6064818; doi:10.3164/jcbn.17-136)
Supplement: Supplemental Table 1 [file jcbn17-136st01.pdf]

Supplemental Table 1. Primer sequences used for mouse genotyping

Primers	Primer sequence (5′ to 3′)
HO-2-F1 (wild-type, forward)	GAGTTGCTGGCTTGGCTTATAG
HO-2-R1 (wild-type, reverse)	TTCCGGTGTAGCTCCGTGGGG
HO-2-F2 (knocked-out, forward)	CAAGGTGAGATGACAGGAGATC
HO-2-R2 (knocked-out, reverse)	GCTTGGGTGGAGAGGCTATTC
