# Supplementary material for: Quantitative imaging mass spectroscopy reveals roles of heme oxygenase-2 for protecting against transhemispheric diaschisis in the brain ischemia
Source: J Clin Biochem Nutr. 2018 Apr 11;63(1):70–9. doi: 10.3164/jcbn.17-136 (PMC6064818; doi:10.3164/jcbn.17-136)
Supplement: Appendices [file jcbn17-136appendix.pdf]

Appendices

Appendix I: Description of RICE Algorithms

RICE consists of five tools and three manual steps (Appendix Fig. 1). These tools are explained in detail below.

Tool 1: RICE_Converter converts ANALIZE7.5 format datasets into CSV files

A raw dataset acquired by a MALDI-IT-TOF mass spectrometer (Shimadzu) was converted to an *ANALYZE7.5* format[(1)] by the data converter function provided by the manufacture (Shimadzu). *ANALYZE7.5* format can accommodate values of mass intensities with a three-dimensional information, namely *x* and *y* coordinate planes and mass-to-charge ratios (*m/z*) (Appendix Fig. 2). ***RICE_Converter*** separates the *ANALYZE7.5* dataset into many CSV files according to specific *m/z* values. Each CSV file is an intensity map with an (*x; y*) coordinate representing a specific *m/z* value. Since the mass resolution (i.e., distance between *m/z* data points) of our experiment is between 0.01638 and 0.024658, a dataset acquired for a range from *m/z* 300 to *m/z* 670 produces approximately 18,060 CSV files.

Tool 2: RICE_Unifier unifies multiple CSV files representing a single metabolite signature

Due to the high mass resolution mentioned above, a single metabolite signature is represented by multiple CSV files. For example, a signature of ATP can be represented by multiple mass values; i.e., *m/z* 505.909, *m/z* 505.931, *m/z* 505.952 and *m/z* 505.994. This necessitates steps to select appropriate mass signals for a single metabolite signature and to unify the information. As shown in Appendix Fig. 3A, a user manually enlarges an appropriate range of the target *m/z* values and selects several candidate mass signals representing a target metabolite. Once these signals are chosen, ***RICE_Unifier*** starts calculating a representative value (e.g., maximum intensity, integrated intensity, or mean intensity) from several CSV formatted intensity map for each spot on *x-y* coordinates. Then it creates a single intensity map (Appendix Fig. 3B).

Tool 3: RICE_ND_Detector tags the spots with non-detectable peaks of a specific metabolite of interest

ANALYZE7.5 format dataset (mentioned in ***Tool 1***) deals with a rectangular array (an *m*-by-*n* map) but not a polygonal array, which is a typical tissue shape such as brain. When we transform a native IMS dataset representing a polygon like a brain, *ANALYZE7.5* constructs a rectangle which mounts the entire polygon inside the array by filling no-tissue pixel with 0 (Appendix Fig. 4A). This causes a problem because computers cannot numerically discriminate the null-values acquired as non-detected (ND) mass peaks of a target metabolite against the null-values created during the data conversion process with the *ANALYZE7.5*. ***RICE_ND_Detector*** is designed to solve this problem. To separate ND-pixel from no-tissue pixel (denoted as green and black pixels respectively in Appendix Fig. 4A), we utilized ion signals of 9-aminoacridine (9-AA) that is a matrix sprayed on the tissue to help ionize endogenous metabolites. Since only ND-pixels, but not no-tissue pixels, should display ion signals of 9-AA, we can exploit the signal (i.e., *m/z* 385.1) to tag ND-pixels (Appendix Fig. 4B). We then assigned the value of 10^{-6} for ND-pixels, while no-tissue pixels were left to be filled with 0. With this, no-tissue pixels were numerically separated from ND-pixels.

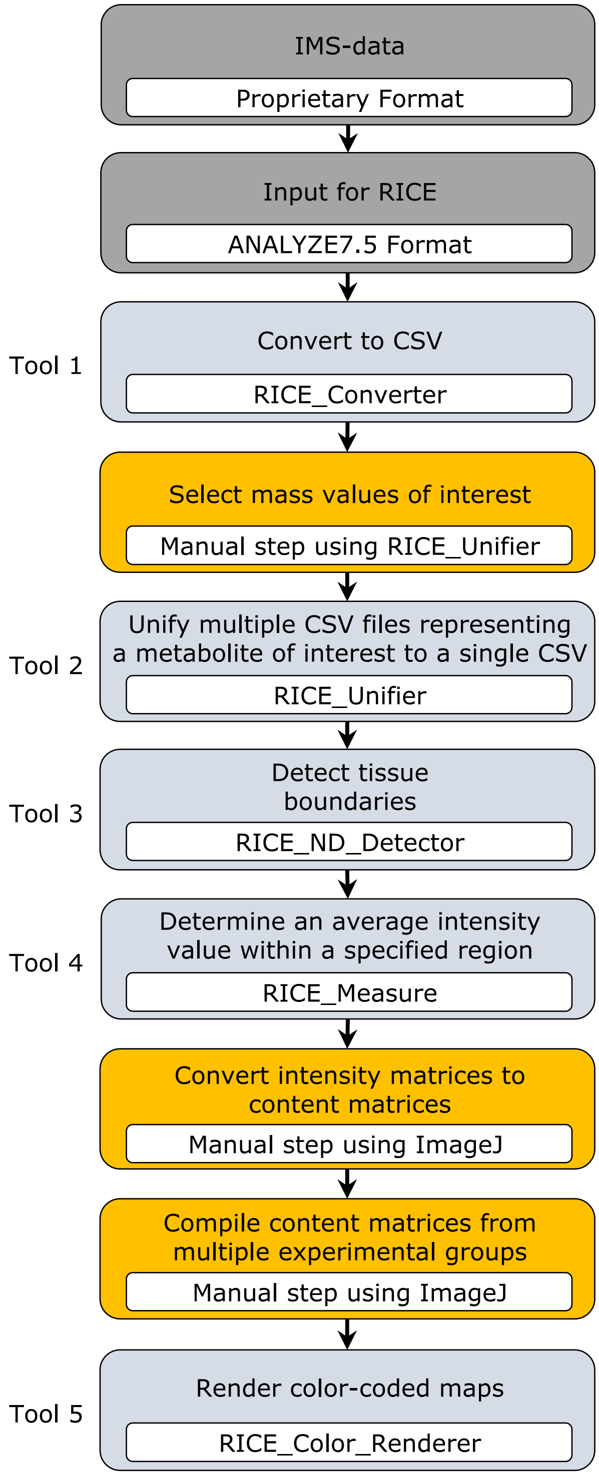

Appendix Fig. 1. Work flow of RICE to generate a color-coded content map from an IMS dataset. The tasks carried out by the tools are indicated with blue background. Manual processes are indicated with orange background.

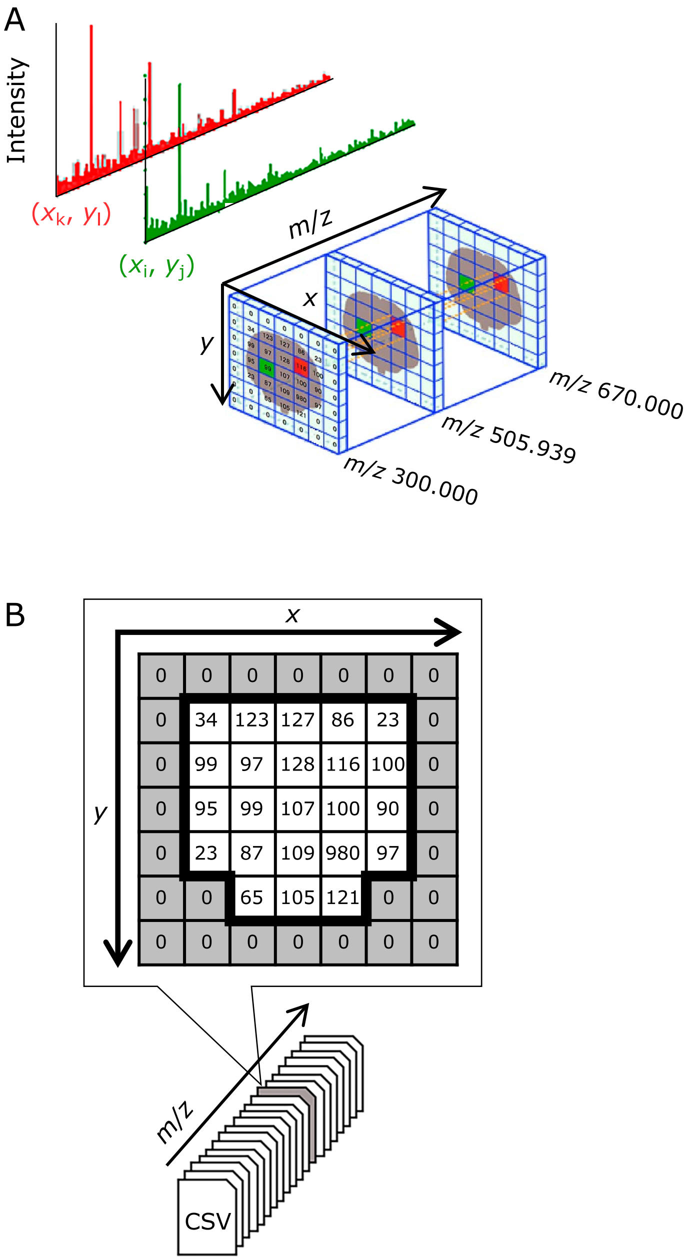

Appendix Fig. 2. Conversion of a proprietary MALDI-IMS dataset to CSV files using ***RICE_Converter***. (A) MALDI-IMS dataset in *ANALYZE7.5* format is a data cube of signal intensity values with 3-dimential information; namely 2 spatial planes of *x* and *y* coordinates and mass-to charge ratios (*m/z*). (B) ***RICE_Converter*** divides the data cube according to specific *m/z* values that ends up producing a series of segmented intensity map as CSV files.

Tool 4: RICE_Measure calculates an average intensity value of a target metabolite within a specified region

To construct apparent content maps for a target metabolite, we used a previously reported method, quantitative imaging mass spectrometry (Q-IMS).[2–6] Briefly, mass signals intensities of a metabolite acquired with MALDI-IMS were converted to apparent contents of a metabolite expressed in an absolute term (tissue content in nmol/g tissue). Apparent content of a metabolite at the ith spot of tissue (C_i) was estimated as follow:

$$C_i = C' \times I_i/\bar{I}$$

where C' denotes the metabolite content of tissue from a contralateral hemisphere determined by the CE/MS, I_i is the maximum intensity among mass spectra in a specified range at the ith spot, and $\bar{I}$ is the median of maximum intensities of a metabolite from all the spots in a contralateral hemisphere.

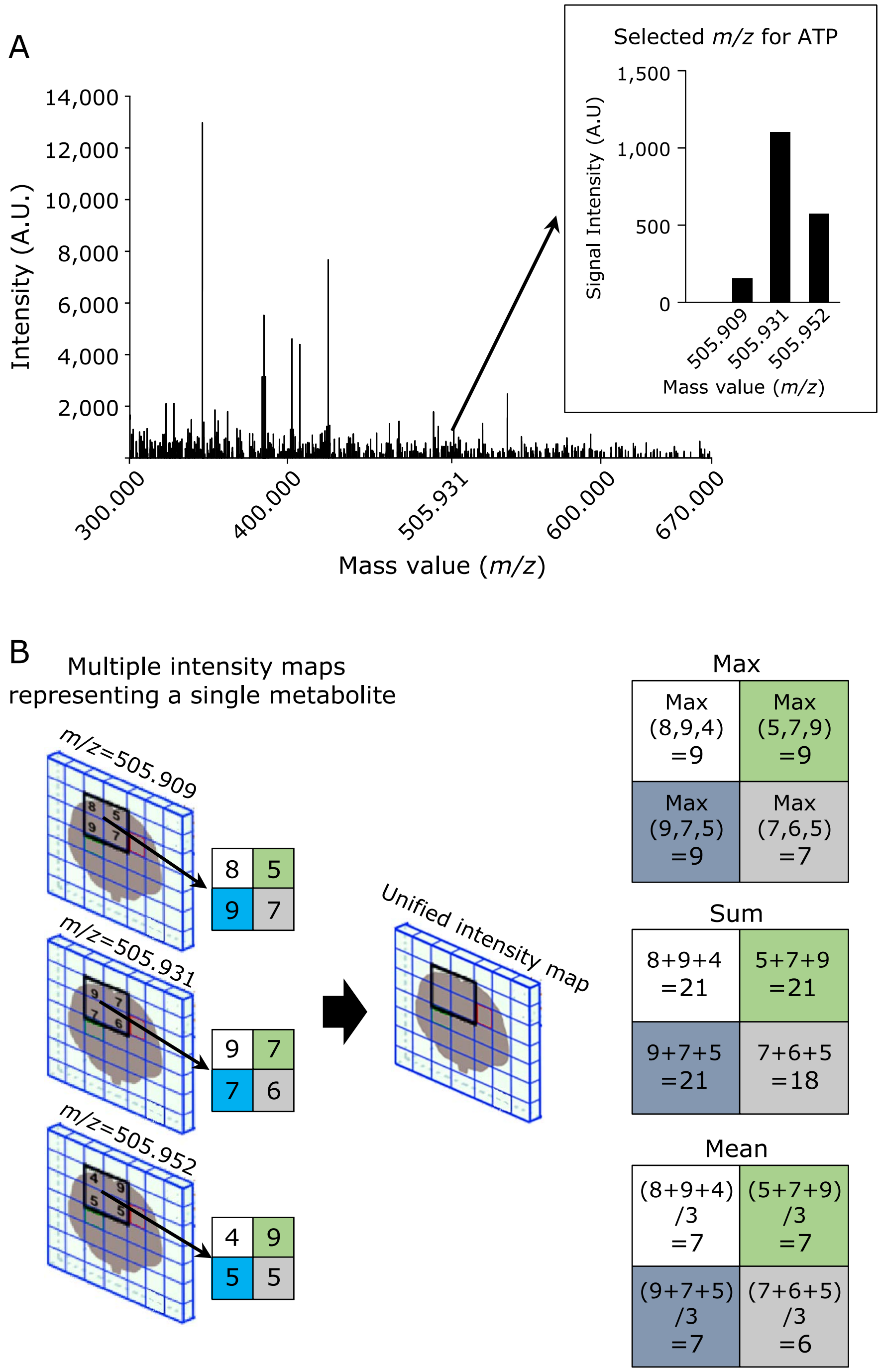

Appendix Fig. 3. ***RICE_Unifier***. (A) The signal intensity values are plotted against the mass values (*m/z*). Note that there are three mass values representing ATP (inset). Signals here are discrete generated by digital modulations with *RICE_Converter*. (B) ***RICE_Unifier*** unifies multiple map representing a single metabolite (e.g., ATP) into a single map. Users can choose three modes of representative values such as maximum, summed and mean intensities.

RICE_Measure calculates an average intensity value ($\bar{I}$) of a target metabolite within a specified region. Finally, ion intensity map of a target metabolite was converted to a content map using ***multiply*** method of ImageJ.

Tool 5: RICE_Color_Renderer renders a color-coded image

RICE_Color_Renderer constructs color-coded content maps by sorting out compiled values into color-coded bins by adapting histogram equalization method (Appendix Fig. 5A).[7–9] Eleven colors easily distinguishable in both RGB and CMYK color scales were used for this tool. First, ***RICE_Color_Renderer*** segregates data points from no-tissue pixels and assigns white color to them. It then assigns dark blue color to ND pixels. Next, it sorts all content values from low to high

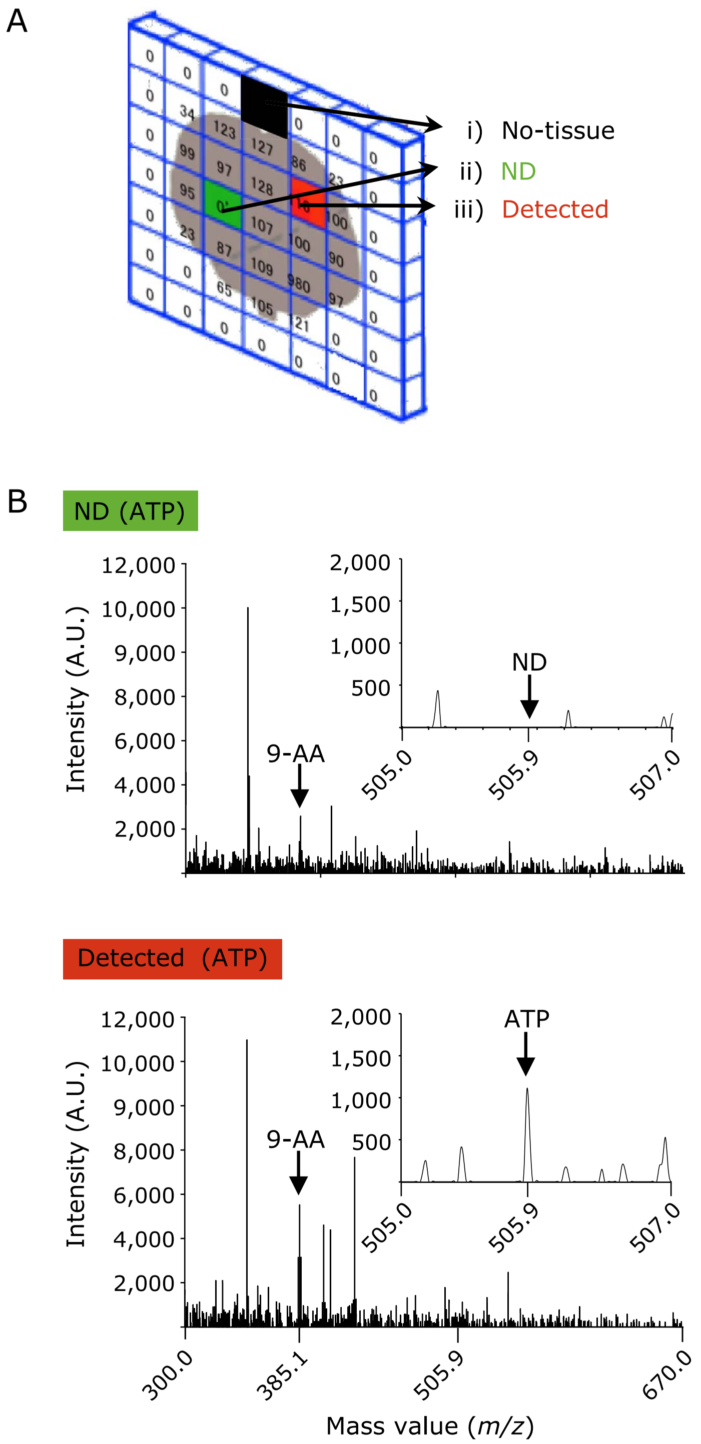

Appendix Fig. 4. ***RICE_ND_Detector***. (A) A model of an intensity map consisting of pixels from 3 distinct regions; i) no tissue regions (black), ii) regions without detectable signals of a target compound, designated as non-detected (ND) region (green), and iii) regions with detectable signals of a target compound (red). (B) Typical mass spectrum of a pixel without ATP signals (top) and that with ATP signals (bottom). The existence of 9-AA peaks around *m/z* 385.1 is used to distinguish no tissue regions from ND regions. These spectra are acquired by Image MS Solution (Shimadzu). They appear continuous signals as opposed to the discrete signals in Appendix Fig. 3A.

numbers and distributes them into 10 bins in a way that each bin contains the same number of content values (Appendix Fig. 5B). These approaches enhance the contrast of rendered images and make it possible to spot spatio-quantitative differences in local metabolism between two groups.

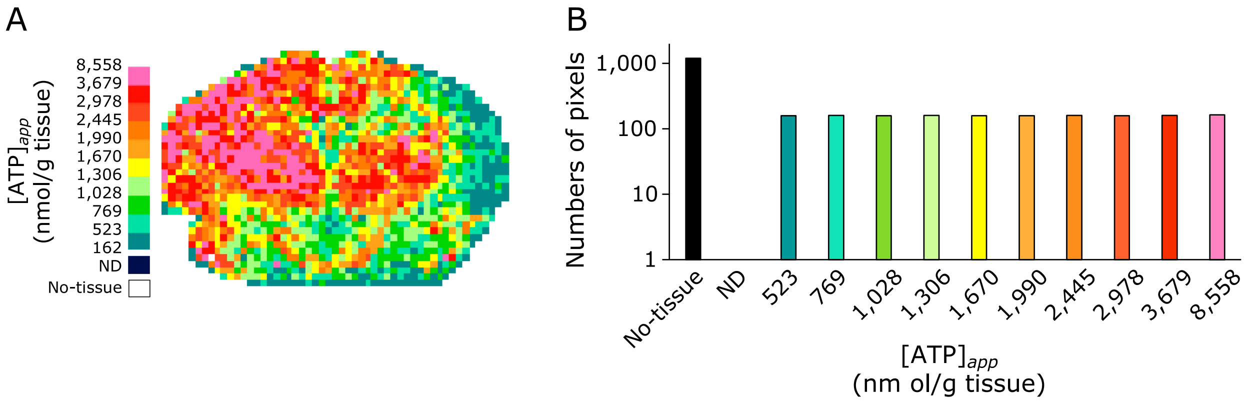

Appendix Fig. 5. Color rendering algorism of ***RICE_Color_Renderer***. (A) Representative color-coded content map of ATP obtained from mouse brain of MCAO-induced focal ischemia model. (B) Histogram of A. Frequency distributions of content values of a metabolite are fitted with *dynamic* interval of the *x*-axis adapting histogram equalization. Same number of content values is allocated to each color level.

Appendix II: Primers to Confirm Heme Oxigenaze-2-null Mice

To identify the genotypes of wild-type and HO-2 null mice. The primers with sequences shown in Supplemental Table 1* were used.

References

1 Robb RA, Hanson DP. ANALYZE: a software system for biomedical image analysis. In: *Proceedings of the First Conference on Visualization in Biomedical Computing, 1990*. 1990; 507–518.

2 Hattori K, Kajimura M, Hishiki T, *et al.* Paradoxical ATP elevation in ischemic penumbra revealed by quantitative imaging mass spectrometry. *Antioxid Redox Signal* 2010; **13**: 1157–1167.

3 Kubo A, Ohmura M, Wakui M, *et al.* Semi-quantitative analyses of metabolic systems of human colon cancer metastatic xenografts in livers of superimmunodeficient NOG mice. *Anal Bioanal Chem* 2011; **400**: 1895–1904.

4 Morikawa T, Kajimura M, Nakamura T, *et al.* Hypoxic regulation of the cerebral microcirculation is mediated by a carbon monoxide-sensitive hydrogen sulfide pathway. *Proc Natl Acad Sci U S A* 2012; **109**: 1293–1298.

5 Yamamoto T, Takano N, Ishiwata K, *et al.* Reduced methylation of PFKFB3 in cancer cells shunts glucose towards the pentose phosphate pathway. *Nat Commun* 2014; **5**: 3480.

6 Yamazoe S, Naya M, Shiota M, *et al.* Large-area surface-enhanced Raman spectroscopy imaging of brain ischemia by gold nanoparticles grown on random nanoarrays of transparent boehmite. *ACS Nano* 2014; **8**: 5622–5632.

7 Verdenet J, Cardot JC, Baud M, Chervet H, Duvernoy J, Bidet R. Scintigraphic image contrast-enhancement techniques: global and local area histogram equalization. *Eur J Nucl Med* 1981; **6**: 261–264.

8 Watrous JD, Alexandrov T, Dorrestein PC. The evolving field of imaging mass spectrometry and its impact on future biological research. *J Mass Spectrom* 2011; **46**: 209–222.

9 Wijetunge CD, Saeed I, Boughton BA, *et al.* EXIMS: an improved data analysis pipeline based on a new peak picking method for EXploring Imaging Mass Spectrometry data. *Bioinformatics* 2015; **31**: 3198–3206.

*See online. https://www.jstage.jst.go.jp/article/jcbn/advpub/0/advpub_17-136/_article
